# An Assessment of Patient Portal Messaging Use by Patients With Multiple Chronic Conditions Living in Rural Communities: Retrospective Analysis

**DOI:** 10.2196/44399

**Published:** 2023-08-01

**Authors:** Fernando L Chivela, Ashley E Burch, Oghale Asagbra

**Affiliations:** 1 Department of Health Services and Information Management East Carolina University Greenville, NC United States; 2 Department of Internal Medicine Brody School of Medicine East Carolina University Greenville, NC United States

**Keywords:** patient portal, multimorbidity, chronic disease, patient messaging, rural, mobile phone

## Abstract

**Background:**

Patient portals can facilitate the delivery of health care services and support self-management for patients with multiple chronic conditions. Despite their benefits, the evidence of patient portal use among patients with multimorbidity in rural communities is limited.

**Objective:**

This study aimed to explore the factors associated with portal messaging use by rural patients.

**Methods:**

We assessed patient portal use among patients with ≥1 chronic diagnoses who sent or received messages via the Epic MyChart (Epic Systems Corporation) portal between January 1, 2015, and November 9, 2021. Patient portal use was defined as sending or receiving a message through the portal during the study period. We fit a zero-inflated negative binomial model to predict portal use based on the patient’s number of chronic conditions, sex, race, age, marital status, and insurance type. County-level characteristics, based on the patient’s home address, were also included in the model to assess the influence of community factors on portal use. County-level factors included educational attainment, smartphone ownership, median income, and primary care provider density.

**Results:**

A total of 65,178 patients (n=38,587, 59.2% female and n=21,454, 32.92% Black) were included in the final data set, of which 38,380 (58.88%) sent at least 1 message via the portal during the 7-year study period. As the number of chronic diagnoses increased, so did portal messaging use; however, this relationship was driven primarily by younger patients. Patients with 2 chronic conditions were 1.57 times more likely to send messages via the portal than those with 1 chronic condition (*P*<.001). In comparison, patients with ≥7 chronic conditions were approximately 11 times more likely to send messages than patients with 1 chronic condition (*P*<.001). A robustness check confirmed the interaction effect of age and the number of diagnoses on portal messaging. In the model including only patients aged <65 years, there was a significant effect of increased portal messaging corresponding to the number of chronic conditions (*P*<.001). Conversely, this relationship was not significant for the model consisting of older patients. Other significant factors associated with increased portal use include being female; White; married; having private insurance; and living in an area with a higher average level of educational attainment, greater medical provider density, and a lower median income.

**Conclusions:**

Patients’ use of the portal to send messages to providers was incrementally related to their number of diagnoses. As the number of chronic diagnoses increased, so did portal messaging use. Patients of all ages, particularly those living in rural areas, could benefit from the convenience and cost-effectiveness of portal communication. Health care systems and providers are encouraged to increase the use of patient portals by implementing educational interventions to promote the advantages of portal communication, particularly among patients with multimorbidity.

## Introduction

### Background

Multimorbidity or multiple chronic conditions are common among adults in the United States and dramatically increase the complexity of patient care [1,2]. Multimorbidity represents the co-occurrence of ≥2 chronic conditions, which may or may not be associated [3-5]. Chronic conditions are characterized as diseases that last 1 or multiple years, require continuous medical attention, or challenge activities of daily living [6]. As of 2022, chronic conditions remain the leading driver of health care costs in the United States [1], and conditions such as heart disease, cancer, diabetes, and obesity are among the most frequent causes of disability and death in adults in the United States [1,7]. For patients living in rural areas, the rate of multimorbidity is higher than that of their urban counterparts [8]; in addition, they experience more barriers to care, including the means to reach and use health care services, such as transportation, health literacy, and health insurance status [8,9]. The continued development and implementation of health information technologies, such as patient portals, can facilitate the delivery of health care services in rural communities by enabling communication between patients and providers without the need for in-person visits.

Since their inception in the late 1990s, patient portals have held the potential to be valuable tools for enhancing the quality of patient involvement in health care [10,11]. Health care systems are able to use patient portals to provide patients with secure web-based access to their personal health information, and the portal allows patients to schedule appointments, receive educational resources, manage medications, and message providers [11]. Current research shows that patients are interested in using patient portals and that the availability of a patient portal is essential to some patients when choosing providers [12,13]. Patient portals improve patient satisfaction, engagement, medication adherence, health status awareness, and communication with providers [14-17]. Furthermore, patient portals have been associated with health care efficiency, resulting in increased profitability of health care organizations [18,19].

Despite the overwhelming evidence of potential benefits, the number of studies investigating the adoption of portals by specific patient groups is limited. Although patient portal use has increased over time, only a fraction of the overall patient population uses patient portals [12,13,15,16,20-24]. Most studies, including systematic reviews conducted between 2015 and 2021, indicate that sociodemographic factors may explain why some patients do not use patient portals [13,15,16,21-25]. These studies suggest that patient characteristics such as age, sex, race, ethnicity, marital status, income, and insurance status constitute barriers to portal use. A limited number of studies have explored patient portal use among patients with >1 chronic condition [12,26,27]. In an investigation of 32,274 urban-dwelling portal adopters, Yamin et al [26] found higher odds of messages sent to physicians’ practices via a portal among patients with ≥2 chronic conditions. Portal use rates among patients with multimorbidity have also increased in recent years because of the Centers for Medicare and Medicaid Services’ implementation of Meaningful Use Stage 2 for electronic health records that requires health care systems to use electronic health records to document care and share health information with other providers and the patient [12].

Increased patient portal use has also been identified among rural and underserved patients with multimorbidities. Among patients aged ≥45 years, Powell and Deroche [27] noted that as the physical distance separating patients from their providers increased, portal logins and use among patients with at least 2 chronic conditions increased. However, most studies examining the relationship between the chronic diagnoses and patient portal use have restricted age ranges; neglected rural patients; or focused on specific, related chronic diseases [12,26]. Only up to 4 chronic conditions were included in these studies, such as hypertension, heart failure, coronary artery disease, and diabetes [12,26,27]. Although previous studies have assessed the use of patient portals, most studies focused on access to medical records, provision of email services, and transactional features [12,14,28,29]. Relatively few studies have assessed the messaging feature of patient portals, but these studies did not explore messaging in the context of multimorbidity or rurality [11,13,15].

Therefore, the current literature on portal use among patients with multimorbidity does not generalize to those with other prevalent chronic conditions such as cancer, obesity, stroke, or arthritis and may not be applicable to younger patients or those living in rural areas. We focused on the messaging feature of the patient portal because of the level of engagement and the potential impact of using this feature in a rural population that could benefit significantly from additional interactions with health care providers that did not require an in-person visit. Our study contributes to the gaps in the literature by analyzing patient portal messaging use among adults living in rural areas with multiple chronic conditions.

### Theoretical Framework

#### Overview

This study draws from the Diffusion of Innovation Theory by Rogers [30-32] and the intersectionality theory by Crenshaw [33,34]. Both theories are appropriate in this context, as they allow us to consider the characteristics of the innovation, the characteristics of the individual patient, and how contextual factors impact the patients’ choice to use the innovation. The innovation of interest in this study was the messaging feature of the patient portal.

#### Diffusion of Innovation Theory

The Diffusion of Innovation Theory suggests that for an innovation to be adopted, it must have several characteristics. First, the innovation must have a relative advantage over other existing methods. Second, it must be compatible and consistent with the users’ values, past experiences, and current needs. Finally, the innovation must also possess a trialability and observability characteristic. In other words, it must be easy to understand by users who can then test the innovation as well as the experience its results. Therefore, how patients with multiple chronic conditions assess the characteristics of the patient portal as an innovation will impact their decision to use the portal.

Patients must assess whether the patient portal offers them an easier and more convenient means to communicate with their providers about their health. If the patient portal provides an advantage over the other methods of communication and if it meets their need for receiving more updated information about their health, then the patient will be more likely to adopt portal use. Given that the patient portal is analogous to using the internet and other applications that are now ubiquitous in society [35], these patients could find the use of the portal consistent with their expectations and experiences. Although trialability and observability can only be achieved over time, it is expected that patients will continue to use the portals if they continue to be satisfied with their interaction. Furthermore, the Diffusion of Innovation Theory suggests that adopters will generally follow the innovation-decision process: knowledge, persuasion, decision, implementation, and confirmation [30]. It is likely that patients who interact frequently with health care providers, such as those with chronic conditions, will become aware of the patient portal and can be persuaded of its benefits. Moreover, patients with multimorbidity may feel a greater need to adopt the patient portal for the purposes of communication with their provider given their health risk. The Diffusion of Innovation Theory also indicates that the innovation-decision process for individuals is influenced by several socioeconomic and community factors, including their education, media exposure, social status, and participation. For individuals with multiple chronic conditions, it is expected that similar factors should be considered as affecting their adoption and use of the patient portal.

#### Intersectionality Theory

It is generally accepted that societal-, community-, and system-level contextual factors can come together to determine a person’s willingness to adopt an innovation. In addition, individual-level characteristics also contribute to the decision to adopt an innovation—in this study, the patient portal. Rogers [31] described the importance of individual characteristics in determining one’s adoption of an innovation. Previous studies have also characterized the importance of socioeconomic and demographic factors in explaining why patients use the patient portal [4,17,36]. They suggest that these patient characteristics could constitute a disadvantage or barrier to using the patient portal.

**Figure 1:**
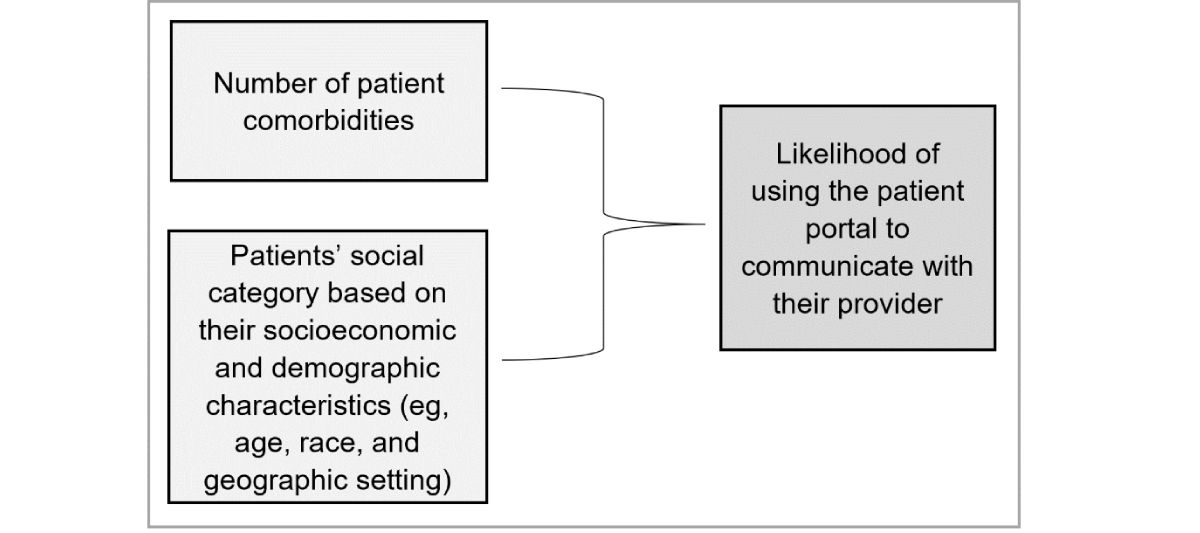
Conceptual model for patient portal use.

The intersectionality theory further adds that there exists interdependencies between social categories to which individuals belong, for example, age, race, sex, marital status, and geographic setting (eg, rural vs urban). The theory suggests that these social categories should not be considered in isolation from each other; rather, social categories intersect to produce complex forms of privilege and inequalities in a society. As such, each individual of a society will have experiences that are shaped by the combined effects of the social categories to which they are affiliated. In the health care literature, the intersectionality theory has been applied to examine systemic barriers that impact the health and well-being of individuals [36-38]. Although this theory is akin to the social determinants of health, which is based on the socioeconomic and demographic characteristics of individuals, it further considers the interaction effects of these social categories and how they combine to impact the overall health of the individual [39]. As such, it is important to consider the combined effects across the social categories to which an individual belongs in determining their choice to use the patient portal (Figure 1).

### Hypotheses

On the basis of the theories discussed in the Theoretical Framework section above, the following hypotheses are presented:

1. Having a greater number of chronic diagnoses will be associated with greater use of the patient portal messaging feature.
2. A patient’s social group, as defined by their socioeconomic and demographic characteristics, will be significantly associated with their decision to use the patient portal messaging feature to communicate with their health care provider.

We anticipate that a patient’s social group, determined by their age, sex, race, marital status, insurance status, education, and income level, will be significantly associated with their use of the patient portal to communicate with their health care provider. On the basis of the literature, we would expect that patients who are female, White, younger, and married will demonstrate higher portal messaging use [13,16]. However, the association between these patient characteristics and portal messaging is yet to be confirmed among rural patients with multimorbidity.

## Methods

### Overview

In this retrospective, cross-sectional study, we investigated the relationship between multimorbidity and patient portal messaging use. We sought to determine the effect of having multiple chronic diagnoses on the use of the messaging feature of the patient portal as a way to communicate with care providers. Chronic diagnoses were based on the International Classification of Diseases, Ninth and Tenth Revisions, Clinical Modification codes for arthritis, asthma, cardiovascular conditions, chronic kidney disease, chronic obstructive pulmonary disease, diabetes, heart diseases, nutritional deficiencies, obesity, sleep disorders, and stroke. These conditions were isolated from the Healthy North Carolina 2030 project, which serves as the population health improvement plan for the North Carolina Division of Public Health [40].

### Ethics Approval

This study (UMCIRB 21-000545) was reviewed and approved by the East Carolina University and Medical Center Institutional Review Board in accordance with federal research regulations.

### Sample

Patient-level data and portal use were extracted through a query from the data warehouse of a hospital system in eastern North Carolina, United States. Although North Carolina contains several urban areas, the primary catchment area of the queried hospital system is eastern North Carolina, a largely rural, agricultural area. Using patients’ home address, they were classified as residing in a rural or primarily suburban county or an urban county based on the population density of the county. The hospital system offers multispecialty care and serves >1 million primarily rural residents. It uses the Epic electronic health record and MyChart patient portal by Epic Systems Corporation [41].

Patients were eligible for inclusion if they (1) were aged between 18 and 89 years, (2) had a home address in North Carolina, (3) had at least 1 of the chronic conditions listed in Table 1, and (4) had received or sent messages via Epic MyChart between January 1, 2015, and November 9, 2021. Patients were excluded if they did not have any of the selected chronic diagnoses or had no portal use.

**Table 1:** Number of people in the final sample diagnosed with a given chronic condition (N=65,178).

Chronic condition	Patients, n (%)
Heart diseases	39,893 (61.21)
Obesity	20,070 (30.79)
Diabetes	17,289 (26.53)
Nutritional deficiencies	13,219 (20.28)
Chronic kidney disease	13,151 (20.18)
Sleep disorders	11,623 (17.83)
Arthritis	11,517 (17.67)
Cardiovascular conditions	11,081 (17)
Stroke	5023 (7.71)
Chronic obstructive pulmonary disease	4877 (7.48)
Asthma	3999 (6.14)

### Definitions

We defined patient portal use as sending or receiving a message through the patient portal. We assigned the level of portal use into two groups: (1) no interaction and (2) interaction. We defined the no interaction group as patients who received ≥1 messages from their providers but did not reply or send messages via the portal. We defined the interaction group as patients who sent ≥1 messages via the portal, regardless of receiving messages from their providers.

### Variables

Variables from the electronic health record included patient age, sex, race, marital status, insurance type, chronic diagnoses, and received or sent messages. Variables from external data sources included county-level educational attainment, smartphone ownership, primary care physician (PCP) density, and median income. We used external data sources to supplement our analysis with relevant information on common public health indicators and targets.

County-level data were obtained from external data sources and merged with the patient-level data based on the patients’ county of residence as recorded in the medical record. Ease of physician access was defined as PCP density (ie, the number of PCPs per 100,000 people at the county level). This variable was obtained from the 2018 Area Health Resource File, released by the Bureau of Health Workforce, a division of the Health Resources and Services Administration [42]. The Health Resources and Services Administration tracks trends in the national health care workforce and releases county-level data sourced annually from the American Medical Association Masterfile, American Dental Association Masterfile, and the Centers for Medicare and Medicaid Services National Provider Identification File.

To obtain data on income, education, and smartphone access, we used the American Community Survey (ACS) 5-year estimates. The ACS is conducted annually by the US Census Bureau to provide detailed information on a broad range of population characteristics to help guide the distribution of federal and state resources. The current data releases of the ACS include 1-year, supplemental, and 5-year estimates. The 5-year estimates were used in this study; these estimates aggregate responses from previous years to balance geographic and temporal fluctuations, thus increasing the statistical reliability of the data for less populated (ie, rural) areas. For this study, we included 3 variables collected by the ACS: smartphone ownership (percentage of households in the county with a smartphone, 2016-2020 ACS), educational attainment (percentage of persons aged ≥25 years with less than a high school diploma, 2013-2017 ACS), and median income (2013-2017 ACS) [43].

### Statistical Analysis

Descriptive statistics characterizing the sample are reported as count (percentage) and mean (SD) as appropriate, unless otherwise noted. To investigate group differences in demographic characteristics between those who sent 0 messages (no interaction group) and those who sent ≥1 messages (interaction group), chi-square tests and 2-tailed *t* tests were used for categorical and continuous variables, respectively.

Several models were evaluated to test the relationship between the number of chronic diagnoses and patient portal use, conceptualized as the number of messages sent by the patient through the portal. Models tested included ordinary least squares, Poisson, and negative binomial models. Fit indices, specifically Akaike and Bayesian information criterion, were compared with each other to identify the model distribution that would provide the best fit for the data while minimizing the potential of inflated variance estimates. The zero-inflated negative binomial model was chosen for the primary analysis as this model demonstrated superior fit indices and could accommodate the prevalence of 0 counts and overdispersion of our dependent variable, number of messages sent (mean 10.92, SD 29.17). In the final model, the dispersion parameter was 3.67 (95% CI 3.60-3.74).

The zero-inflated negative binomial model simultaneously estimates 2 equations. The first equation is based on a negative binomial distribution and was used to estimate the count of portal messages sent by patients in the portal interaction group (ie, patients who sent at least 1 message via the portal). The second equation provided by the model is based on a logistic distribution and is used to estimate the odds of having excess zeros beyond what would be expected by random chance. In other words, this second equation is the probability that a patient would not use the portal (ie, that the patient would send 0 messages through the portal) based on the evaluated predictors.

The model was adjusted for individual- and community-level predictors. Individual-level predictors included patients’ age, sex, race, insurance status, and marital status. County-level covariates were based on the participants’ county of residence and included percentage of the population with an educational attainment of a high school diploma or greater, percentage of the population reporting smartphone ownership, PCP density, and median income.

All missing values were excluded from multivariate analysis. Analyses were performed using SPSS statistics (version 28.0; IBM Corp) and SAS (version 9.5; SAS Institute). All tests are 2 tailed. *P* values <.05 were considered statistically significant.

## Results

### Participants

Given the location of the hospital in eastern North Carolina, approximately all the participants resided in a rural or suburban county (64,656/65,178, 99.2%). Table 2 provides sample characteristics for the full sample (N=65,178), for participants who did not send any messages during the study period (no interaction group: n=26,798, 41.12%), and for those who sent at least 1 message during the study period (interaction group: n=38,380, 58.88%). On the basis of the univariate analysis, patients who interacted with the portal were more likely to be female; White; younger; married; have private insurance; and live in an area with a higher average level of educational attainment, more prevalent smartphone ownership, higher PCP density, and a higher median income.

**Table 2:** Demographic factors and social determinants of health for the full sample, those who sent no messages (no interaction), and those who sent ≥1 messages (interaction)^a^.

Demographics		Full sample (N=65,178)	No interaction (n=26,798, 41.12%)	Interaction (n=38,380, 58.88%)	*P* value	
**Sex, n (%)**						<.001
	Female	38,587 (59.2)	14,898 (55.59)	23,689 (61.72)		
	Male	26,591 (40.8)	11,900 (44.41)	14,691 (38.28)		
**Race, n (%)**						<.001
	Black	21,454 (32.92)	10,313 (38.48)	11,141 (29.03)		
	White	41,840 (64.19)	15,741 (58.74)	26,099 (68)		
	Other	1139 (1.75)	395 (1.47)	744 (1.94)		
	Missing	745 (1.14)	349 (1.3)	396 (1.03)		
Age (years), mean (SD)		57.09 (15.33)	59.40 (15.23)	55.48 (15.19)	<.001	
**Marital status, n (%)**						<.001
	Single	27,824 (42.69)	12,681 (47.32)	15,143 (39.46)		
	Married	37,104 (56.93)	13,995 (52.22)	23,109 (60.21)		
	Missing	250 (0.38)	122 (0.46)	128 (0.33)		
**Insurance, n (%)**						<.001
	Private	27,597 (42.34)	9313 (34.75)	18,284 (60)		
	Government	19,820 (30.41)	9779 (36.49)	10,041 (33.2)		
	Uninsured	2101 (3.22)	1008 (3.76)	1093 (3.6)		
	Other	1660 (2.55)	793 (2.96)	867 (2.9)		
	Missing	14,000 (21.48)	5905 (22.04)	8095 (21.09)		
**Educational attainment (%), mean (SD)**						<.001
	High school diploma or greater	84.93 (5.51)	83.86 (5.65)	85.68 (5.29)		
**Smartphone ownership (%), mean (SD)**						<.001
	Household with a smartphone	78.11 (6.89)	77.07 (6.93)	78.84 (6.77)		
**PCP^a^ density, mean (SD)**						<.001
	PCP/100,000 population	60.43 (27.98)	56.20 (27.05)	63.37 (28.23)		
**Median income (US $), mean (SD)**						<.001
	Median household income	44,488.82 (6955.39)	43,534.86 (6882.22)	45,154.91 (6928.67)		

^a^PCP: primary care physician.

### Portal Use

Table 3 presents the results of the zero-inflated negative binomial model. Model estimates, SEs, and incidence rate ratios (the exponentiation of the regression coefficient) or odds ratios for the logit model were reported. As expected, patients diagnosed with more chronic conditions were more likely to use the portal than those diagnosed with fewer chronic conditions. The relationship between chronic disease and portal use was exponential, with additional diagnoses resulting in an increased use of the portal messaging feature. The expected sent message count for patients diagnosed with 2 chronic diseases was 1.57 times higher than that for a patient with 1 chronic condition. The sent message count for a patient with ≥7 chronic diseases was considerably higher, approximately 11 times the message count of a patient with 1 chronic condition. Additional significant predictors of increased portal use were female sex; White race; being married; having private insurance; younger age; and living in an area with higher educational attainment, greater PCP density, and a lower median income.

**Table 3:** Zero-inflated negative binomial results predicting the number of sent messages.

Parameter			Count model							Logit model				
			Estimate (SE; 95% CI)		*P* value		IRR^a^		Estimate (SE; 95% CI)			*P* value		Odds ratio (95% CI)
**Number of chronic diseases (reference: 1)**														
	7	2.3917 (0.2134; 1.9734 to 2.8100)		<.001		10.93		0.0719 (0.2807; −0.4782 to 0.6221)			.80		1.07 (0.62 to 1.86)	
	6	2.0868 (0.1655; 1.7624 to 2.4111)		<.001		8.06		0.8547 (0.1612; 0.5387 to 1.1707)			<.001		2.35 (1.71 to 3.22)	
	5	1.5392 (0.1219; 1.3003 to 1.7781)		<.001		4.66		0.2448 (0.1430; −0.0355 to 0.5251)			.09		1.28 (0.97 to 1.69)	
	4	1.1668 (0.0893; 0.9918 to 1.3418)		<.001		3.21		0.4185 (0.1114; 0.2002 to 0.6368)			<.001		1.52 (1.22 to 1.89)	
	3	0.7715 (0.0598; 0.6543 to 0.8887)		<.001		2.16		0.2995 (0.1006; 0.1024 to 0.4966)			.003		1.35 (1.11 to 1.64)	
	2	0.4497 (0.0354; 0.3804 to 0.5191)		<.001		1.57		0.1586 (0.0965; −0.0306 to 0.3477)			.10		1.17 (0.97 to 1.42)	
**Sex (reference: male)**														
	Female	0.2605 (0.0211; 0.2191 to 0.3018)		<.001		1.30		−0.5168 (0.0738; −0.6614 to −0.3722)			<.001		0.60 (0.52 to 0.69)	
**Race (reference: White)**														
	Black	−0.5409 (0.0234; −0.5867 to −0.4951)		<.001		0.58		−0.0319 (0.4233; −0.8616 to 0.7978)			.94		0.97 (0.42 to 2.22)	
	Other	−0.0510 (0.0782; −0.2042 to 0.1021)		.51		0.95		1.1497 (0.0762; 1.0002 to 1.2991)			<.001		3.16 (2.72 to 3.67)	
**Marital status (reference: single)**														
	Married	0.1541 (0.0211; 0.1127 to 0.1955)		<.001		1.17		−0.5348 (0.0750; −0.6818 to −0.3877)			<.001		0.59 (0.51 to 0.68)	
**Insurance status (reference: insured)**														
	Government	−0.0434 (0.0218; −0.0862 to −0.0006)		.047		0.96		1.1913 (0.0916; 1.0117 to 1.3709)			<.001		3.29 (2.75 to 3.94)	
	Uninsured	−0.4225 (0.0516; −0.5236 to −0.3215)		<.001		0.66		1.3464 (0.1715; 1.0102 to 1.6826)			<.001		3.84 (2.75 to 5.38)	
	Other	−0.1816 (0.0578; −0.2948 to −0.0683)		.002		0.83		1.1321 (0.1620; 0.8146 to 1.4496)			<.001		3.10 (2.26 to 4.26)	
Age			−0.0108 (0.0013; −0.0133 to −0.0083)		<.001		0.99		0.0375 (0.0025; 0.0326 to 0.0423)			<.001		1.04 (1.03 to 1.04)
Education			0.0326 (0.0046; 0.0236 to 0.0415)		<.001		1.03		−0.13 (0.0201; −0.1695 to −0.0906)			<.001		0.88 (0.84 to 0.91)
PCP^b^ density			0.0027 (0.0007; 0.0014 to 0.0041)		<.001		1.00		0.0091 (0.0039; 0.0015 to 0.0167)			.02		1.01 (1.00 to 1.02)
Smartphone			−0.0062 (0.0037; −0.0135 to 0.0010)		.09		0.99		−0.1162 (0.0138; −0.1431 to −0.0892)			<.001		0.89 (0.87 to 0.91)
Median income			−0.0058 (0.0015; −0.0088 to −0.0028)		<.001		0.99		0.0247 (0.0060; 0.0130 to 0.0364)			<.001		1.03 (1.01 to 1.04)
Age × number of chronic diseases			−0.0040 (0.0005; −0.0050 to −0.0031)		<.001		1.00		N/A^c^			N/A		N/A
Dispersion			3.6683 (0.0364; 3.5977 to 3.7402)		N/A		N/A		N/A			N/A		N/A

^a^IRR: incidence risk ratio.

^b^PCP: primary care physician.

^c^N/A: not applicable.

Given that the incidence of chronic disease increases as a person ages and the direction of the significant relationship between age and portal use in our sample, we added a parameter to explore the interaction of chronic disease and age on portal use. This interaction term was significant in our model. Results from the excess zero model (logit model) are presented in Table 3. The outcome of the multivariate analysis supports the findings from the univariate analysis.

### Model Robustness

**Figure 2:**
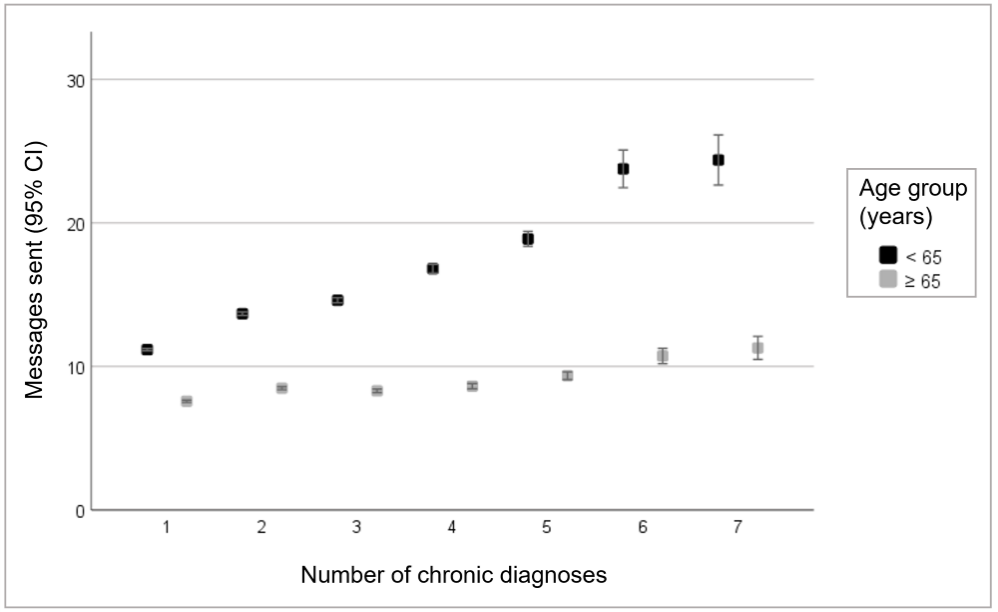
Number of messages sent based on the number of diagnosed chronic diseases by age group.

A robustness check using 2 models was performed to verify the interaction effect of age and the number of diagnoses on portal use. In the first model, only patients aged <65 years were included. Excluding patients aged ≥65 years from this model allows us to obtain more accurate estimates of the relationship between chronic disease and portal use, as portal use by older patients did not appear to be significantly influenced by the number of diagnoses and including them in the comprehensive model likely caused an underestimation in the results. In the second model, we excluded younger patients (those aged <65 years) to confirm the results from our main analysis, specifically, that portal use was not related to the number of diagnoses for the older patients in our sample. Results are presented in Table 4. The robustness check revealed results consistent with our main analysis, verifying the importance of age when considering the relationship between multimorbidity and the use of the electronic messaging feature of patient health portals. Figure 2 shows the predicted values for the number of sent messages from the negative binomial model based on the number of chronic diagnoses and age.

**Table 4:** Zero-inflated negative binomial results predicting the number of sent messages.

Parameter			Aged <65 years						Aged ≥65 years				
			Estimate (SE; 95% CI)		*P* value		IRR^a^		Estimate (SE; 95% CI)		*P* value		IRR
**Number of chronic diseases (reference: 1)**													
	7	2.7288 (0.301; 2.1388 to 3.3188)		<.001		15.31		0.4592 (0.8011; −1.1110 to 2.0294)		.57		1.58	
	6	2.3191 (0.2282; 1.8719 to 2.7663)		<.001		10.17		0.5559 (0.6439; −0.7060 to 1.8179)		.39		1.74	
	5	1.7701 (0.1703; 1.4363 to 2.1039)		<.001		5.87		0.3066 (0.5031; −0.6795 to 1.2927)		.54		1.36	
	4	1.3227 (0.1247; 1.0782 to 1.5671)		<.001		3.75		0.2639 (0.3784; −0.4778 to 1.0056)		.49		1.30	
	3	0.9165 (0.0834; 0.7531 to 1.0800)		<.001		2.50		0.0784 (0.253; −0.4175 to 0.5744)		.76		1.08	
	2	0.5073 (0.0467; 0.4158 to 0.5987)		<.001		1.66		0.1402 (0.1321; −0.1186 to 0.3991)		.29		1.15	

^a^IRR: incidence risk ratio.

## Discussion

### Principal Findings

In this study, we assessed the relationship between multimorbidity and patient portal messaging use, controlling for several individual- and community-level characteristics known to influence portal use. We found that patients diagnosed with multiple chronic conditions were significantly more likely to use patient portal messaging than those diagnosed with fewer chronic conditions. Consistent with previous studies, the findings of our study indicate that there are still disparities in patient portal use based on demographic factors such as age, race, and marital status. Notably, we found that insurance coverage and several county-level factors, namely, PCP density, education, income, and access to technology, are significantly associated with patient portal use for sending messages. We describe the underlying meaning of our findings in the discussion below and provide recommendations for future research.

Our analysis showed that as the number of chronic diagnoses increased, so did patient portal messaging use; however, this relationship was driven primarily by younger patients. The Diffusion of Innovation Theory suggests that the perceived advantage offered by an innovation, such as a patient portal, is an important factor in determining its use [30]. Studies have found that patients use portals if they perceive them to offer a relative advantage over existing practices such as calling or traveling for an inpatient visit with their provider [44]. Given that patients with multimorbidity tend to have complex health needs, rely on a network of providers, and consume more medical services than those without chronic diseases [45-47], theory suggests that they would be able to recognize the benefits and anticipated advantages that portal messaging could offer [48,49]. However, it is possible that such advantages are not yet fully realized by older patients or that they may focus more on the portal’s incompatibility with their values or experiences. Additional qualitative or survey exploration should be conducted for the specific reasons that portal adoption is delayed, as this may provide clarification and identify actions that could be taken to improve engagement (eg, one-on-one coaching).

Younger patients have been identified as more frequent users of patient portals than their older counterparts [12,13,17]. The slower adoption of portals by older patients is because of several factors, including physical barriers, lack of experience, interest, and access to the necessary technologies [50]. However, portal use among older patients is increasing because of patients’ overall comfort with technology, chronic condition management needs, and social motivation [16,50-53]. Although less frequent use of the patient portal by older patients has been associated with medical care avoidance [54], previous research conducted within our patient population suggests that younger patients in our region may be more likely to avoid care [55]. In addition, all patients included in our sample received or sent at least 1 portal message, suggesting that they had a visit or some interaction with a care provider during the study period. Therefore, the truly medically avoidant population is unlikely to be represented in our sample. From the perspective of the Diffusion of Innovation Theory, health care systems seeking to increase the adoption and use of patient portals across different demographic groups are encouraged to implement educational interventions to promote the advantages of portal communication, particularly among patients with multimorbidity [44].

We also found that significant predictors of increased patient portal messaging use included demographic factors such as sex, marital status, race, educational attainment, provider density, and income. These findings can be viewed through the lens of the intersectionality theory, which suggests that the combined individual characteristics of the patient must be considered when evaluating disparities and how they might constitute an advantage or disadvantage to the patient. Hence, our findings suggest that female, younger, married, and White patients and patients who live in areas with a higher educational attainment, greater PCP density, and lower median income are more likely to communicate with their providers via the patient portal. These findings align with those of the previous studies examining sociodemographic differences in patient portal use. Similar results highlight disparities among patient portal users, with rural, male, older, single, uninsured, publicly insured, and racial minority patients being disadvantaged [12,13,15,16,20-24,56,57]. Some studies, however, showed mixed results regarding sex and age differences in patient portal use [11,58,59]. A systematic review conducted to evaluate the use and impact of access to electronic medical records among patients with type 2 diabetes found increased portal adoption and use rate among older male patients [58]. They also noted that women aged >65 years were less likely to access electronic health–related services than older men, who reported being more familiar with the internet through employment [58].

In our sample, Black patients sent fewer messages than White patients. This finding is consistent with previous studies reporting that patient portal use is commonly lower among Black, Hispanic, and Asian patients [20,57,60]. Patients reporting good internet access, higher income, and living in urban areas have been identified as more frequent users of patient portals [61,62]. In contrast, we found increased portal use among patients living in areas with a lower median income. It is plausible that patients with lower income view the portal as a way to communicate with their providers that does not require additional resources. For example, patients with low-income status may have employment that does not provide paid time off, they may not have reliable transportation for an in-person visit, or they may not have the financial resources to pay copays and deductibles. Therefore, our findings may indicate that, for these patients, the benefit of an in-person visit may be outweighed by the convenience and cost-effectiveness of communication through the patient portal.

### Limitations

This study offers important insights for health care administrators and researchers. However, there are several limitations that must be considered. First, this study was limited to patients of a major medical center in the eastern region of North Carolina; therefore, the results may not be generalizable to all individuals in the United States and other countries. Second, given that most of the counties in the region are rural, the results may be more relevant to individuals living in rural areas who typically have some resistance to innovation and fewer resources available to allow them to access and use the relevant technology. Third, this study was limited by the unavailability of certain variables that have been shown to contribute to the use of patient portals [63]. For example, variables that measure the associated costs of using the technology and the availability of technical support were not controlled for in this study. Future studies should consider extending their models to include these and other additional variables when examining the association between the patient portal use and multimorbidity. Fourth, the sample for this study was restricted to patients diagnosed with physical conditions selected a priori based on recommendations from the Healthy North Carolina 2030 project. Consequently, mental health diagnoses were not included in our data set; however, given the known association between mental health diagnoses and physical multimorbidity, future studies should explore mental health conditions as an element of multimorbidity and its impact on portal messaging use [64-67]. Fifth, this study primarily assessed portal messages sent by patients to providers, which does not explore potential biases in providers’ engagement with patients via the patient portal. It is possible that providers may be less likely to message older patients believing that older patients may be less comfortable with technology compared with younger patients [23,68]. Finally, this study investigated use of the messaging feature of the patient portal. It is important to note that several other features are available in many patient portals (eg, access to test results and transactional services), and the use of these features may or may not correlate with use of the messaging feature.

Notwithstanding, this study contributes to the health information technology and patient portal use literature by emphasizing the characteristics of patients with multimorbidity that are more likely to use patient portals for sending messages. It also highlights the challenges that key stakeholders must consider when encouraging patients with multimorbidity to use the patient portal. By better understanding how the characteristics of individual patients with multimorbidity influence their use of the patient portal while also considering the combined effects of these characteristics, administrators and policy makers may gauge their outreach decisions as well as how they allocate resources to encourage the use of patient portals to achieve their maximum potential.

### Conclusions

Managing multimorbidity is challenging for the patient, the provider, and the larger health care system. Electronic patient portals are a valuable tool for enhancing patient engagement and have the capacity to improve health care delivery by allowing patients to access their medical records, schedule and manage appointments, and communicate directly with their care providers. In our study, patients’ use of the portal to send messages to providers was incrementally related to their number of diagnoses. We found that as the number of chronic diagnoses increased, so did patient portal use; however, this relationship was driven primarily by younger patients. As a person ages, their likelihood of experiencing multimorbidity increases, whereas their capacity for adopting new technologies may decline. Given these considerations, tailored interventions, such as the use of peer advisors, coaches, and educational videos shown in clinic waiting areas, could be used to encourage and advocate for the use of patient portals among older patients. Furthermore, patients of all ages living in rural areas could benefit from the convenience and cost-effectiveness of portal communication. Future studies should investigate portal use among patients with multimorbidity in the context of additional social determinates of health, and prospective studies should be designed to test interventions to improve portal use in this population.

## Abbreviations

ACS: American Community Survey
PCP: primary care physician

## Data Availability

The data used in this study are not publicly available. The data set was obtained from East Carolina University (ECU) Health, and privacy and security concerns prevent the redistribution of data to investigators outside of the institutional review board–approved study team. Researchers can apply for access to the individual-level data set by submitting a request to the ECU Health Center for Research and Grants.
